# Implantation

**Published:** 1888-12

**Authors:** C. Cutler Smith

**Affiliations:** Ilian, N. Y.


### IMPLANTATION.

C. CUTLER SMITH, ILIAN, N. Y.

My attention was first called with special interest to the new and astonishing method of implanting teeth about two years ago. After a careful examination of the subject, I was emboldened to enter upon its practice myself. Since last October I have performed the operation of implantation twelve times, and I take great pleasure in reporting that in every instance the results so far as developed have been entirely satisfactory. Leave was taken to follow the methods of the best operators, and every precaution was used to preserve antiseptic conditions. In the selection and preparation of the teeth used painstaking attention should be given to every detail, in order that the process of healing may easily and promptly be accomplished. The operation itself is properly in the line of surgery, and naturally requires a thorough knowledge of the subject in hand.

In all my operations the staple has been used, and the result has been that every tooth implanted has been retained firmly in its place, while unfavorable symptoms have not appeared. While this report extends over a period of less than a year, yet my experience confirms the conviction I had previously reached that implantation can be made permanently beneficial. In the cases under my care, the patients have had complete use of the new members, almost entirely unattended by pain or inconvenience. Implantation, if its future is what it to-day promises to be, will become one of the grandest and most beneficent features of dentistry.

The first dentists extracted teeth; next came those who repaired and saved diseased teeth, or replaced them by artificial ones. The third step was the introduction of crown work, in which the root is saved; and of bridge-work, in which two roots are made to do the work of three or more. Is it not reasonable to assume that the next higher step has been attained in implantation, where the diseased tooth is replaced by a sound one, which takes its place as if it has been an own child, and not an adopted member of the family?

My own faith in the permanent success of this new method is strong, and I trust that, after a suitable period has elapsed, I shall be able to report the continued success which has thus far followed the implantations I have undertaken. The mere fact that unfavorable symptoms have not appeared after the lapse of several months is in itself a promise that the results will be permanently beneficent.

A patient who has the use of the new teeth even for a period of not longer than two or three years would be well repaid, but the experience thus far recorded indicates that where operations have been performed, after due care has been taken in respect to the conditions of the patient, and the selection of suitable teeth, and with needful and antiseptic precautions, implantation is permanent. When this is fully established by the lapse of time, dental science will have been shown to have taken one of the mightiest strides in its history.

Implantation has already proved a benefit to the profession, and a boon to suffering and disfigured faces. It has stimulated the best minds in the profession to discover methods for establishing the permanency of the new operation, and it has already had important results in elevating and broadening the scope of our work.

